# Determinants of Consumption of Vegetables among the Spanish Population: A Cross-Sectional Study

**DOI:** 10.3390/foods12214030

**Published:** 2023-11-05

**Authors:** María Orosia Lucha-López, César Hidalgo-García, Ana Carmen Lucha-López, Sofía Monti-Ballano, Sergio Márquez-Gonzalvo, Loreto Ferrández-Laliena, Héctor José Tricás-Vidal, José Miguel Tricás-Moreno

**Affiliations:** 1 Unidad de Investigación en Fisioterapia, Spin off Centro Clínico OMT-E Fisioterapia SLP, Universidad de Zaragoza, Domingo Miral s/n, 50009 Zaragoza, Spain; orolucha@unizar.es (M.O.L.-L.); smonti@unizar.es (S.M.-B.); 724250@unizar.es (S.M.-G.); lferrandez@unizar.es (L.F.-L.); hjtricas@unizar.es (H.J.T.-V.); jmtricas@unizar.es (J.M.T.-M.); 2 Unidad de Investigación en Fisioterapia, Universidad de Zaragoza, Domingo Miral s/n, 50009 Zaragoza, Spain

**Keywords:** vegetables, dietary habits, demographic factors, social factors, BMI, Spain

## Abstract

The consumption of vegetables is one of the fundamentals of a healthy diet. The purposes of the present study were to describe the frequency of consumption of vegetables in the general Spanish population and to explore the relations between the consumption of vegetables and sex, age, cohabitation circumstances, educational level, and body mass index (BMI). Methods: An analytical cross-sectional study was accomplished based on data from the European Health Survey in Spain (2020). Results: A total of 20,745 (52.1% women) subjects with a median age of 54 years old were included. Only 2.8% of them ate vegetables at least three times a day. The adjusted generalized linear model showed that being a woman increased the odds of consuming vegetables at least three times a day by 1.666 times (*p* < 0.001). Not cohabiting as a couple decreased the odds by 0.783 (*p* < 0.001). Having studied at a university increased the odds by 1.812 times (*p* < 0.001) and possessing a certificate of higher education by 1.408 (*p* = 0.030). Being overweight decreased the odds by 0.924 (*p* = 0.006). For every additional year of age, the odds of consuming vegetables at least three times a day increased by 1.3% (*p* < 0.001). Conclusions: The vast majority of the general Spanish population did not consume an optimal amount of vegetables. Women, people with higher levels of education, and older individuals reported having a more frequent intake of vegetables. Not cohabiting as a couple and being overweight were related to a less frequent intake of vegetables.

## 1. Introduction

In the International Scientific Symposium about biodiversity and sustainable diets, celebrated in 2010 at the Food and Agriculture Organization of the United Nations headquarters in Rome, sustainable diets were defined as “diets with low environmental impact which contribute to food and nutrition security and to healthy life for present and future generations” [1]. 

The consumption of vegetables is one of the fundamentals of a healthy and sustainable diet. Fruits, vegetables, and whole grains are key dietary components for maintaining a healthy body weight, protecting bodies against certain cancers [2], and reducing risk for diabetes, heart disease, stroke, and other chronic diseases [3]. A high intake of vegetables is one of the healthy components of traditional diets [4]. Different health outcomes have been described for fruits and vegetables, but the majority of studies investigating vegetable consumption have not investigated vegetable consumption independently of fruit consumption; thus, separate research for these different food classes has been recommended [5]. Green leafy vegetables appear to be the most beneficial foods for the prevention of major chronic diseases and cardiovascular diseases [6]. 

The World Health Organization recommends the intake of at least ≥5 servings of about 80-g (≥400 g equivalent) of fruits and vegetables (excluding potatoes and other starchy tubers) per day [7]. A large proportion of the world population does not meet these recommendations [8]. In Europe, more than half of the countries have an intake of fruits and vegetables lower than 400 g per day [3]. Particularly, in eastern Europe countries, the average intake is less than 300 g per day [3]. Thus, it is necessary to make recommendations to the population regarding healthy plant-based foods. Diets that reduce or eliminate the consumption of animal products maximize the favorable impact of a diet on human, animal, and environmental health [3].

The Lancet Commission on Healthy Diets for Sustainable Food Systems proposes an intake of 300 g/day of vegetables and states that a universal, healthy, and sustainable diet must be based on an increase in the consumption of vegetables, fruits, whole grains, legumes, and nuts [9].

According to the food-based dietary guidelines from the Spanish Society of Community Nutrition, a sustainable diet must include three portions of vegetables per day [10], considering a portion to comprise between 150 and 200 g of net and raw vegetables. In addition, the Spanish Society of Community Nutrition recommends including three or four servings, or pieces, of a variety of fruit per day [10,11]. Thus, the recommendations in Spain regarding fruits and vegetables’ consumption seem to be higher than the World Health Organization’s recommendations. In this regard, there is a need to consider the fact that populations in Southern Europe have shown the highest odds of consuming vegetables in Europe [12], maybe due to the high availability, affordability, and traditional consumption of fruits and vegetables in this area [13,14].

Never consuming vegetables was linked, in 2014, with a cost of EUR 139,000 for every 1000 persons in the Spanish health system. This charge might be avoidable with adequate education and prevention [15]. 

Though some studies have analyzed the social and demographic determinants of vegetable consumption, it appears that the relation between vegetable consumption and sociodemographic factors is not obvious. Being a woman [16,17] and a more educated individual [18,19] have been related, with some consistency, to more vegetable consumption. However, older adults have been identified as larger consumers of vegetables in some studies [20,21] but as lesser consumers in others [22], and being married has shown associations to a better intake of vegetables [23] or to a poorer one [23,24]. An increased intake of vegetables is recommended for obesity prevention but, as far as we know, there are no recent studies in this regard in representative samples of the general population. 

Performing new epidemiological studies has been highlighted as a need for clarifying these issues [25]. Unhealthy diets are characterized by high intakes of added sugar, trans-fat, saturated fat, and sodium and low intakes of vegetables, fruits, nuts and seeds, fish, and whole grains [26]. Unhealthy diets are consumed by a significant amount of the world population, a fact which contributes to premature death and to a substantial increase in the incidence of obesity, coronary heart disease, stroke, and diabetes, among others [9]. Recognizing the actual food consumption patterns and their influencing factors based on reliable surveys can favor the design of prevention programs promoting healthy and sustainable diets [7]. 

The purposes of the present study were to describe the frequency of consumption of vegetables in the general Spanish population and to explore the relations between the consumption of vegetables and sex, age, cohabitation circumstances, educational level, and body mass index. The present study is one of scarce studies dedicated only to vegetable consumption and not to fruit and vegetable consumption taken together, and it analyzes the most recent data available from the general Spanish population on the issue. 

## 2. Materials and Methods 

### 2.1. Study Design

This study accessed data from the European Health Survey in Spain (2020), which is a national survey performed to provide information on the health and wellbeing of the general Spanish population. The Spanish version of the 2020 European Health Surveys was approved by the Commission Regulation (European Union) 2018/255 of the 19 February 2018. The data were anonymous, non-confidential, and publicly accessible. An analytical cross-sectional observational study was performed. 

### 2.2. Setting

Experienced interviewers from the National Statistical Institute (INE) performed personal or telephone interviews between July 2019 and July 2020. 

The recruitment of the sample was randomly performed, stratifying the selection by region, census sections, and family homes, and by the ultimate election of one participant in each family home with the Kish grid [27].

### 2.3. Participants

Data were registered from 22,072 individuals. The inclusion criteria were as follows: participants had to range in age from 15 to 104 years and be a non-institutionalized Spanish resident. 

In this study, the individuals with no responses in some of the variables of interest were excluded. The final sample was composed of 20,745 individuals. 

### 2.4. Data Sources

For the present study, we extracted data on the following:

- Sex: Men/Women.
- Age: Registered in years.
- Cohabitation circumstances: with a spouse, with a domestic partner, and no cohabitation as a couple.
- Educational level: cannot read or write, incomplete primary studies (has attended school for less than 5 years), complete primary education, first stage of secondary education, complete secondary education, vocational education and training, certificate of higher education, and university studies.
- Body Mass Index (BMI): Height and weight were reported by the participants. The BMI was divided into the categories defined by the World Health Organization (WHO) (underweight: <18.5 kg/m^2^; normal: 18.5–24.9 kg/m^2^; overweight: 25.0–29.9 kg/m^2^; obese: ≥30 kg/m^2^) [28]. The self-reported height and weight of the participants have been deemed as valid, with moderate to good agreement in comparison with direct measurements [29].
- How often do you eat vegetables and salads? The individuals only had to tick one of the following options: never, less than one time/week, one or two times/week, three times/week, between four and six times/week, and one or more times/day. How many? [17,30,31]. To describe the data the next categories were created as follows: never, less than one time/week, one or two times/week, three times/week, between four and six times/week, one time/day, two times/day, three times/day, and between four and eight times/day. The vegetables and salads’ group refers to herbaceous horticultural plants that can be consumed raw (salads) or cooked (vegetables). The potato, due to its high starch content, is not included in this group [11].

### 2.5. Statistical Methods

Sex, cohabitation, educational level, BMI, and the consumption frequency of vegetables and salads were explained offering the absolute frequencies and the percentages in each category. Age was described with the median, 25th percentile (Q1), and 75th percentile (Q3), because it was not normally distributed according to the Kolmogorov–Smirnov test.

Univariable generalized linear models (GLM) were constructed to model vegetable consumption as a function of each of the following independent variables separately: sex, age, cohabitation, educational level, and BMI.

One multivariable, generalized, linear model was constructed to model vegetable consumption as a function of all the independent variables together. The models calculated the main effects using multinomial as the distribution and cumlogit as the link function. The hybrid method was applied for parameter estimation. Pearson chi-square was used as the scale parameter method. 

In the generalized linear models, the categories three times/day and between four and eight times/day from the variable consumption frequency of vegetables and salads were grouped together in a new category, which was named at least three times/day. These frequencies were put together to create a category that approached the recommendations from the Spanish Society of Community Nutrition (at least three times/day) [10]. 

The statistical significance was established at *p* value < 0.05. SPSS 25.0 for Mac was used for the calculations.

## 3. Results

A descriptive study of the sample is presented in Table 1. We analyzed data from 20,745 individuals, with a median age of 54 (Q1: 41.0–Q3: 68.0) years. A total of 52.1% of the sample were women and 47.9% of the sample were men. A total of 27.4% (*n* = 5684) of the sample consumed vegetables between four and six times a week, which was the category with the highest percentage. A total of 26.7% of the sample (*n* = 5541) ingested vegetables one time a day, 14.8% (*n* = 3080) of the sample two times a day, 2.3% (*n* = 471) of the sample three times a day, and 0.5% (*n* = 109) of the sample between four and eight times a day. Thus, only 2.8% (*n* = 580) of the total sample ate vegetables at least three times a day. In summary, 44.3% (*n* = 9201) of the sample consumed vegetables one or more times per day.

In the crude analysis, being a woman, not cohabiting as a couple, being overweight, and having an increased age were found to be predictive of vegetables consumption (Table 2). Being a woman increased the odds of consuming vegetables at least three times a day by 1.677 times (*p* < 0.001). Not cohabiting as a couple decreased the odds by 0.772 in relation to cohabiting with a spouse (*p* < 0.001). Being overweight decreased the odds by 0.892 times in relation to being of a normal weight (*p* = 0.004). For every additional year of age, the odds of consuming vegetables at least three times a day increased by 1.0% (*p* < 0.001).

In the multivariable, generalized linear model, the dispersion coefficient was 0.691 and the omnibus test was significative (*p* < 0.001). The generalized linear model parameter estimates are given in Table 3. Being a woman increased the odds of consuming vegetables at least three times a day by 1.666 times (*p* < 0.001). Not cohabiting as a couple decreased the odds by 0.783 in relation to cohabiting with a spouse (*p* < 0.001). Having studied at a university increased the odds by 1.812 times in relation to not being able to read or write (*p* < 0.001). Possessing a certificate of higher education increased the odds by 1.408 times in relation to not being able to read or write (*p* = 0.030). Being overweight decreased the odds by 0.924 times in relation to being of a normal weight (*p* = 0.006). For every additional year of age, the odds of consuming vegetables at least three times a day increased by 1.3% (*p* < 0.001).

## 4. Discussion

In this study, based on data from 20,745 individuals, with a median age of 54 years old and a percentage of women of 52.1%, only 2.8% (*n* = 580) of the sample ate vegetables at least three times a day. This low percentage of people who seem to be complying with the recommendations is of crucial importance because plant-based diets, established on legumes, whole grains, fruits, vegetables, seeds, and nuts, have been proven to ameliorate the health of the general population, with their effects being mediated through bioactive compounds [32], and a higher intake of fruits and vegetables has been associated with a decreased mortality in all causes [33]. This is especially important in the elderly because this type of diet has been proven to be associated with a lower risk of frailty [34]. An inadequate vegetables consumption has been observed, as derived from the Food and Agriculture Organization data on the vegetables consumed per capita per day from 1990 to 2010, in 99.6% of the general population studied across 187 countries [21], in 94.1% of United States-based adults between 20 and 85 years of age [35], in 53.0% of the Chinese population between 18 and 64 years old [36], in 74.1% of the adult population of the Brasília federal district, in Brazil [37], in 98.5% of Australian adults aged 25 years or more [22], in 98% of adolescents in the United States [38], and in 82% of adolescents in Jordania [39]. Only four countries from the worldwide population studied from 1990 to 2010 achieved an optimal mean intake of vegetables (Zimbabwe, Botswana, Swaziland, and Greece) [21]. In Spain, the mean vegetable intake in 2010 for adults ≥20 years of age was between 150 and 174.9 g/d, clearly below recommendations [21]. Socio-demographic and cultural factors, environmental conditions, and worldwide tendencies have been identified as barriers to the consumption of fruits and vegetables [40]. Between the worldwide and environmental tendencies, increasing urbanization may be related to the low consumption of vegetables. Living in an urban environment distances people from primary food production, which can have a negative impact on the availability of sufficient fruits and vegetables, especially for the most disadvantaged people in said urban environment [4]. In adults, the socio-demographic and cultural factors behind mayor vegetable consumption range from taste and pleasure to individual cognitions about nutritional and culinary knowledge and a higher appreciation of health, increased time to prepare meals, and an increased availability and reduced cost of vegetables [5].

In a recent study across six European countries (Belgium, Finland, Spain, Greece, Hungary, and Bulgaria), only 13.5% of the parents in low socio-economic status areas complied with the World Health Organization’s guidelines regarding fruit and vegetable intake [30]. Access and exposure to a range of fruits and vegetables at home is important for the development of preferences for these foods, as is the parental model related to healthy diet and physical activity [4,22]. School-based nutrition interventions consisting in activities that provide scholars with information on healthy eating in an enjoyable format have also proven to be useful [41]. The relation between the access to vegetables and their consumption is not so clear [42], both in terms of how an explicit non-availability of vegetables affects consumption and in cases in which access is guaranteed. In any case, an inadequate intake of vegetables in a diet is a serious public health concern, and the recent data in our study confirm the issue. It has been shown previously that, when compared to fruits, there are even more difficulties to increasing vegetable intake [43], including a lack of time or of the skills needed to cook [44], or a less attractive smell, taste, or texture compared to fruits [45]. A lack of knowledge about recommendations might be a limitation to enhancing vegetable consumption as well; thus, considering the current situation, it seems that educational campaigns on vegetables consumption are required [22]. 

A variety of sociodemographic factors have been shown to influence eating patterns. In our study, being a woman increased the odds of consuming vegetables at least three times a day by 1.666 times (*p* < 0.001), confirming sex-related differences in vegetable intake [24,46,47]. This relation might be mediated by the fact that women have shown to have more knowledge of an adequate intake [22]. Women have been linked to more health-conscious eating [48], though it has been observed as well that very often women’s food choices are related to a concern regarding their appearance more than health [49]. 

Not cohabiting as a couple decreased the odds of consuming vegetables at least three times a day in relation to cohabiting with a spouse. These results agree with previous studies that have demonstrated that married people consumed more vegetables and fruits [50] but are in contrast with others [24,51]. This relation might be mediated as well by the fact that married people have shown to have more knowledge of an adequate intake [22]. 

Having studied at a university or possessing a certificate of higher education increased the odds of consuming vegetables at least three times a day in relation to not being able to read or write. The association between educational level and vegetable consumption is similar to that shown previously: a higher educational level attained is associated with eating the recommended quantity of vegetables [52]. Health literacy has been shown to be a mediator between social status and health [53]. Health literacy is defined as “people’s knowledge, motivation and competences to access, understand, appraise and apply health information in order to make judgments and take decisions in everyday life concerning healthcare, disease prevention and health promotion to maintain or improve quality of life during the life course” [54]. In fact, having a better knowledge of an adequate vegetable intake has been related to higher vegetable consumption [22], and a higher educational level has been related to better nutritional knowledge [43]. In Spanish workers aged between 18 and 65 years old, health care professionals, scientists, engineers, and teachers had the highest vegetable consumption [17]. Thus, the association between a higher educational level attained and the alignment with vegetable intake recommendations may serve as an indicator of social inequality in health [47]. Public health programs aimed at enhancing health literacy, especially among people with lower education levels, might be useful for meeting healthy diet consumption recommendations in the general population. 

In the present study, being overweight decreased the odds of consuming vegetables at least three times a day in relation to being of a normal weight. A low vegetable intake has been previously related, in a prospective study of a large cohort of individuals in North America, to weight gain [55], and a higher BMI has been related to the under-consumption of vegetables in United States resident adults [35]. Lower vegetable and fruit intakes have been related previously to overweight and obesity in the general population between 18 and 65 years old in Türkiye [56]. However, in our study, obesity was not related to vegetable intake, neither to a higher nor a lower one, more than being of a normal weight. This circumstance might be due to the fact that the proportion of obese individuals that eat the recommended amount of vegetables is similar to that of normal-weight individuals but that obese individuals increase their total energy intake with other, high-fat and caloric foods, such as red meat [57]; this is considering, as well, the fact that, in the entire sample, the proportion of individuals eating the recommended amount of vegetables was very low. 

For every additional year of age, the odds of consuming vegetables at least three times a day increased by 1.3% (*p* < 0.001). In the Chinese general population aged between 18 and 64 years old, increasing age was also a protective factor for an adequate vegetable intake [36]. In United States resident adults between 20 and 85 years of age, vegetable intake was also related to age, where, among male and female residents aged 51 years and over, 6.1% and 10.8% met the recommendations, respectively, while, in subjects between 20 and 30 years of age, only 1.1% of male residents and 0.3% of female residents met the recommendations [35]. Considering that food consumption tends to decrease with age, possibly due to a variety of factors including the use of medications, masticatory difficulty, deterioration of physiological functions, especially gastrointestinal function, decreased activity, reduced income, or living alone [58], this association is of special relevance. The study by Giuli et al. showed that in elderly subjects aged 65 years or over, from Central Italy, the most-consumed food was raw vegetables [58]. In Italy, as in Spain, the traditional gastronomy is based on the Mediterranean diet, characterized by a higher consumption of plant-based foods, including vegetables. There is growing evidence showing that the elderly living in Mediterranean zones might better hold traditional, healthy dietary habits than the younger inhabitants [14,59]. 

### Limitations

The cross-sectional design of this study allows for associations to be established between the factors, but avoids implication of causality. All the methods for measuring dietary intake have a degree of bias, especially the self-reported estimated intakes [60], though these are widely used due to the wide quantity and variety of information that might be recorded through them and their low cost. The frequency of vegetable consumption has been registered, but the portion size of the servings or the number of servings each time has not been registered. In order to compare the vegetable intake in our sample to the recommendations, it has been supposed that every time that the individuals in the sample have consumed vegetables, they have ingested a serving of around 200 g. A serving of around 200 g of vegetables has been stablished as a typical portion size in Spain [61]. This comparability has been used in previous studies [31,62]. A similar short-form food frequency questionnaire has been shown to be an effective method of assessing diet quality [31]. However, it is necessary to consider that this fact may generate a bias in the net amount of vegetables consumed. Thus, though our results do not show clear inconsistencies, they should be considered with caution. The large sample size allows national representativeness, but external validity might be compromised for other nations. 

## 5. Conclusions

In short, the evidence presented in this study suggests that the vast majority of the general Spanish population did not consume an optimal amount of vegetables. Women, people with higher levels of education, and older individuals reported having a more frequent intake of vegetables. Not cohabiting as a couple and being overweight were related to a less frequent intake of vegetables.

## Figures and Tables

**Table 1:** Descriptive characteristics of the study sample.

Characteristics (*n* = 20,745)	Total	Men	Women
**Sex**	***n* (%)**		
Men	9947 (47.9)		
Women	10,798 (52.1)		
**Cohabitation**	***n* (%)**	***n* (%)**	***n* (%)**
With a spouse	10,280 (49.6)	5342 (53.7)	4938 (45.7)
With a domestic partner	395 (1.9)	196 (2.0)	199 (1.8)
No cohabitation as a couple	10,070 (48.5)	4409 (44.3)	5661 (52.4)
**Educational level**	***n* (%)**	***n* (%)**	***n* (%)**
Cannot read or write	151 (0.7)	30 (0.3)	121 (1.1)
Incomplete primary studies	1708 (8.2)	695 (7.0)	1013 (9.4)
Complete primary education	3821 (18.4)	1766 (17.8)	2055 (19.0)
First stage of secondary education	4989 (24.0)	2607 (26.2)	2382 (22.1)
Complete secondary education	2698 (13.0)	1341 (13.5)	1357 (12.6)
Vocational education and training	1441 (6.9)	722 (7.3)	719 (6.7)
Certificate of higher education	1797 (8.7)	962 (9.7)	835 (7.7)
University studies	4140 (20.0)	1824 (18.3)	2316 (21.4)
**BMI**	***n* (%)**	***n* (%)**	***n* (%)**
Underweight	405 (2.0)	82 (0.8)	323 (3.0)
Normal	8871 (42.8)	3553 (35.7)	5318 (49.2)
Overweight	8107 (39.1)	4620 (46.4)	3487 (32.3)
Obese	3362 (16.2)	1692 (17.0)	1670 (15.5)
**Consumption frequency of vegetables and salads**	***n* (%)**	***n* (%)**	***n* (%)**
Never	121 (0.6)	84 (0.8)	37 (0.3)
Less than one time/week	327 (1.6)	215 (2.2)	112 (1.0)
One or two times/week	1795 (8.7)	1122 (11.3)	673 (6.2)
Three times/week	3617 (17.4)	1968 (19.8)	1649 (15.3)
Between four and six times/week	5684 (27.4)	2741 (27.6)	2943 (27.3)
One time/day	5541 (26.7)	2419 (24.3)	3122 (28.9)
Two times/day	3080 (14.8)	1186 (11.9)	1894 (17.5)
Three times/day	471 (2.3)	172 (1.7)	299 (2.8)
Between four and eight times/day	109 (0.5)	40 (0.4)	69 (0.6)
**Age**	***n* (%)**	***n* (%)**	***n* (%)**
From 15 to 18 years	622 (3.0)	305 (3.1)	317 (2.9)
From 19 to 29 years	1631 (7.9)	802 (8.1)	829 (7.7)
From 30 to 39 years	2517 (12.1)	1225 (12.3)	1292 (12.0)
From 40 to 49 years	3934 (19.0)	2013 (20.2)	1921 (17.8)
From 50 to 59 years	3741 (18.0)	1870 (18.8)	1871 (17.3)
From 60 to 69 years	3474 (16.7)	1683 (16.9)	1791 (16.6)
From 70 to 79 years	2873 (13.8)	1311 (13.2)	1562 (14.5)
From 80 to 89 years	1635 (7.9)	644 (6.5)	991 (9.2)
From 90 to 99 years	313 (1.5)	94 (0.9)	219 (2.0)
From 100 to 104 years	5 (0.02)	0 (0.0)	5 (0.05)
	**Median (Q1–Q3)**	**Median (Q1–Q3)**	**Median (Q1–Q3)**
**Age (years)**	54.0 (41.0–68.0)	53.0 (40.0–67.0)	55.0 (41.0–70.0)

**Table 2:** Univariable, generalized linear models of consumption frequency of vegetables and salads at least three times/day. Parameter estimates.

Univariable Analysis. Dependent Variable: Consumption Frequency of Vegetables and Salads at Least Three Times/Day	B	Crude Exp(B)	Wald 95% Confidence Interval for the Exp(B) Lower Bound–Upper Bound.	Wald Chi-Square Statistic	*p* Value
**Sex**					
Men		Reference category			
Women	0.517	1.677	1.509–1.863	92.354	<0.001
**Cohabitation**					
With a spouse		Reference category			
With a domestic partner	−0.209	0.811	0.562–1.172	1.242	0.265
No cohabitation as a couple	−0.259	0.772	0.698–0.854	25.148	<0.001
**Educational level**					
Cannot read or write		Reference category			
Incomplete primary studies	−0.150	0.861	0.542–1.396	0.400	0.527
Complete primary education	−0.172	0.842	0.535–1.324	0.555	0.456
First stage of secondary education	−0.369	0.692	0.441–1.086	2.565	0.109
Complete secondary education	−0.130	0.878	0.556–1.386	0.312	0.576
Vocational education and training	−0.166	0.847	0.531–1.351	0.485	0.486
Certificate of higher education	−0.120	0.887	0.559–1.409	0.257	0.612
University studies	0.220	1.246	0.792–1.958	0.906	0.341
**BMI**					
Underweight	−0.098	0.906	0.698–1.176	0.549	0.459
Normal		Reference category			
Overweight	−0.114	0.892	0.826–0.963	8.467	0.004
Obese	−0.012	0.988	0.893–1.093	0.052	0.819
**Age (years)**	0.010	1.010	1.008–1.011	137.781	<0.001

**Table 3:** Multivariable, generalized linear model of consumption frequency of vegetables and salads at least three times/day. Parameter estimates.

Multivariable Analysis. Dependent Variable: Consumption Frequency of Vegetables and Salads at Least Three Times/Day	B	Adjusted Exp(B)	Wald 95% Confidence Interval for the Exp(B) Lower Bound–Upper Bound.	Wald Chi-Square Statistic	*p* Value
**Sex**					
Men		Reference category			
Women	0.510	1.666	1.583–1.753	386.488	<0.001
**Cohabitation**					
With a spouse		Reference category			
With a domestic partner	−0.103	0.902	0.752–1.082	1.232	0.267
No cohabitation as a couple	−0.244	0.783	0.744–0.824	89.155	<0.001
**Educational level**					
Cannot read or write		Reference category			
Incomplete primary studies	−0.072	0.931	0.686–1.264	0.211	0.646
Complete primary education	0.004	1.004	0.744–1.355	0.001	0.981
First stage of secondary education	0.042	1.043	0.772–1.408	0.075	0.784
Complete secondary education	0.296	1.345	0.992–1.823	3.636	0.057
Vocational education and training	0.264	1.302	0.954–1.778	2.768	0.096
Certificate of higher education	0.342	1.408	1.034–1.918	4.718	0.030
University studies	0.594	1.812	1.340–2.450	14.915	<0.001
**BMI**					
Underweight	−0.130	0.878	0.731–1.055	1.925	0.165
Normal		Reference category			
Overweight	−0.079	0.924	0.873–0.978	7.533	0.006
Obese	0.019	1.019	0.947–1.098	0.261	0.610
**Age (years)**	0.013	1.013	1.011–1.014	247.901	<0.001

## Data Availability

Data are available in the “European Health Survey in Spain (EESE) year 2020” at https://www.ine.es, accessed on 10 January 2022.
